# Effects of remote transitional care on stroke patients: a meta-analysis

**DOI:** 10.3389/fneur.2026.1829581

**Published:** 2026-06-05

**Authors:** Lili Bai, Yenan Shen, Huafen Gu

**Affiliations:** Department of Neurology, First People’s Hospital of Linping District, Hangzhou, Zhenjiang, China

**Keywords:** depression, meta-analysis, quality of life, randomized controlled trial, readmission, stroke, telemedicine, transitional care

## Abstract

**Background:**

Stroke is a leading cause of adult disability and mortality worldwide. Although advances in acute stroke care have improved survival rates, challenges remain in the post-discharge transitional period. Remote transitional care has emerged as a promising service model to improve outcomes in stroke patients, but its effectiveness remains unclear.

**Objective:**

This study aimed to systematically evaluate the effects of remote transitional care on quality of life, depressive symptoms, and readmission rates in stroke patients through meta-analysis.

**Methods:**

PubMed, Cochrane Library, Embase, and Web of Science were systematically searched from inception to February 2026. Randomized controlled trials evaluating the effects of remote transitional care on stroke patients were included. Two reviewers independently performed study selection, data extraction, and risk of bias assessment using the Cochrane ROB 2.0 tool. Meta-analyses were conducted using RevMan 5.4 software. Mean differences or standardized mean differences were calculated for continuous outcomes, and risk ratios were calculated for dichotomous outcomes, with 95% confidence intervals for all effect estimates.

**Results:**

Six randomized controlled trials were included. Meta-analysis demonstrated that remote transitional care significantly improved depressive symptoms in stroke patients (SMD = −0.28, 95% CI −0.44 to −0.12, *p* = 0.0005, *I*^2^ = 0%). However, no significant benefits were observed for quality of life (SMD = 0.04, 95% CI −0.02 to 0.10, *p* = 0.19, *I*^2^ = 4%) or readmission rates (RR = 1.21, 95% CI 0.82 to 1.78, *p* = 0.34, *I*^2^ = 0%). Sensitivity analyses confirmed the robustness of these findings. Risk of bias assessment rated five studies as having “some concerns” and one study as “high risk.”

**Conclusion:**

Remote transitional care significantly improves depressive symptoms in stroke patients, although its effects on quality of life and readmission rates remain uncertain. Future high-quality, large-sample randomized controlled trials are needed to further validate its effectiveness and to explore optimal intervention modalities and implementation strategies.

## Introduction

Stroke remains one of the leading causes of adult disability and mortality worldwide, imposing a substantial burden on patients, families, and healthcare systems (1, 2). Advances in acute stroke treatments have led to declining mortality rates; however, survivors often experience varying degrees of functional impairment requiring long-term rehabilitation and support after hospital discharge (3–5). Concurrently, decreasing lengths of acute hospital stay have rendered the post-discharge transition period a vulnerable phase in the continuum of stroke care (6, 7).

Transitional care (TC) is defined as a set of actions designed to ensure the continuity and coordination of healthcare as patients transfer between different levels of care or from hospital to home, encompassing discharge planning, follow-up, medication management, rehabilitation guidance, and health education (8). For stroke patients, high-quality transitional care is crucial for preventing complications, reducing readmission rates, promoting functional recovery, and improving quality of life (8, 9).

In recent years, the rapid advancement of telemedicine technologies has provided new modalities for delivering transitional care. Remote transitional care utilizes information technologies such as telephone follow-up, mobile health applications, remote monitoring devices, and video conferencing to transcend geographical barriers, enabling patients to continue receiving professional care after discharge (10, 11). This model is particularly beneficial for patients in underserved areas with limited access to healthcare resources, offering the potential to improve service accessibility and reduce healthcare costs (11, 12).

Although numerous randomized controlled trials have investigated the effects of remote transitional care on stroke patients, the findings have been inconsistent. Some studies have demonstrated that remote interventions can improve functional outcomes and quality of life (13, 14) and reduce the risk of hospital readmission (14), while others have failed to observe significant benefits (15, 16). Furthermore, considerable heterogeneity exists across studies in terms of intervention intensity, follow-up duration, and outcome measures, limiting the generalizability and clinical applicability of individual study findings (17).

Currently, systematic reviews specifically examining the effects of remote transitional care on stroke patients remain insufficient, particularly those incorporating recent high-quality pragmatic randomized controlled trials. The selection of quality of life, depressive symptoms, and readmission rates as primary outcomes is grounded in their established clinical relevance to post-stroke transitional care. Quality of life serves as a comprehensive patient-reported outcome capturing physical, psychological, and social domains of recovery. Depressive symptoms are highly prevalent after stroke and are closely linked to lower quality of life, poorer functional outcomes, and increased healthcare utilization. Readmission rate directly reflects the effectiveness of transitional care in preventing complications and reducing unnecessary hospitalizations. Together, these three outcomes encompass both patient-centered well-being and healthcare system performance, providing a balanced evaluation of remote transitional care. Therefore, this study aims to systematically evaluate the effects of remote transitional care on quality of life, depressive symptoms, and readmission rates among stroke patients through meta-analysis, with the goal of providing evidence-based guidance for clinical practice and health policy development.

## Methods

### Study design and registration

This systematic review and meta-analysis was reported in accordance with the Preferred Reporting Items for Systematic Reviews and Meta-Analyses (PRISMA) guidelines (18). The study protocol was registered with the International Prospective Register of Systematic Reviews (PROSPERO) under registration number CRD420261336592. As this study involved secondary analysis of published literature, ethical approval was not required.

### Search strategy

We systematically searched four electronic databases: PubMed, Cochrane Library, Embase, and Web of Science, from their inception to February 2026. The search strategy combined Medical Subject Headings (MeSH) terms with free-text keywords related to three core concepts: “stroke,” “transitional care,” and “telemedicine.” Additionally, we manually screened the reference lists of included studies to identify potentially eligible studies not captured by the electronic search.

### Inclusion and exclusion criteria

Studies were included based on the PICOS framework: (1) Participants: adults aged ≥18 years with a confirmed diagnosis of stroke (ischemic or hemorrhagic); (2) Interventions: remote transitional care, defined as ongoing professional care, follow-up, or rehabilitation support delivered via telecommunication technologies (e.g., telephone, video conferencing, mobile health applications, remote monitoring devices) after hospital discharge; (3) Comparators: usual care or other non-remote forms of post-discharge care; (4) Outcomes: studies reporting at least one of the following outcomes—depressive symptoms, quality of life, or readmission rate; (5) Study design: randomized controlled trials. Exclusion criteria included non-randomized studies, observational studies, conference abstracts, case reports, reviews, and studies for which full text was unavailable.

### Study selection and data extraction

Two reviewers independently performed study selection. Titles and abstracts were first screened to exclude obviously irrelevant records, followed by full-text assessment of potentially eligible studies to determine final inclusion. Disagreements were resolved through discussion or consultation with a third reviewer. Data extraction was conducted independently by two reviewers using a standardized data extraction form. Extracted information included: first author, publication year, country, sample size, patient characteristics, intervention details (content, duration, and frequency), comparator details, follow-up time points, outcome measures, and measurement instruments. For missing data, we attempted to contact corresponding authors via email.

Among the three studies included in the meta-analysis of depressive symptoms, the measurement instruments were as follows: the Center for Epidemiologic Studies Depression Scale (CES-D) was used in two studies, and the Southampton Stroke Self-Management Questionnaire (SSSMQ) depression subscale was used in one study [Markle-Reid et al. (13) reported both CES-D and SSSMQ]. The CES-D cutoff score for clinically significant depressive symptoms varied: Linder et al. used a cutoff of ≥10, while Markle-Reid et al. (13) reported mean CES-D scores without specifying a cutoff for caseness. One additional study [Allen et al. (17)] reported CES-D data but was excluded from the meta-analysis because only covariate-adjusted mean differences were provided without group-specific means or standard deviations, precluding calculation of effect sizes. Regarding severity grading, none of the three included studies reported the proportion of patients with mild, moderate, or severe depression based on CES-D score ranges. None of the included studies systematically classified post-stroke depression according to diagnostic criteria such as DSM-5 or ICD-11; instead, all relied on symptom scales. This variability in assessment methods and the lack of uniform severity grading limit the comparability of depression outcomes across studies, which we have acknowledged as a limitation.

### Risk of bias assessment

The methodological quality of included randomized controlled trials was assessed using the Cochrane Risk of Bias tool version 2.0 (ROB 2.0). The assessment covered five domains: bias arising from the randomization process, bias due to deviations from intended interventions, bias due to missing outcome data, bias in measurement of the outcome, and bias in selection of the reported result. Two reviewers independently evaluated each domain, rating them as “low risk,” “some concerns,” or “high risk.” The overall risk of bias was determined based on the domain-level assessments. Results are presented as risk of bias summary figures.

### Statistical analysis

Meta-analyses were performed using RevMan 5.4 software. For dichotomous outcomes, risk ratios with 95% confidence intervals were calculated. For continuous outcomes, mean differences were calculated when the same measurement instruments were used across studies; standardized mean differences with 95% confidence intervals were calculated when different instruments were used to measure the same construct. Heterogeneity was assessed using the *χ*^2^ test and *I*^2^ statistic: *I*^2^ ≤ 50% indicated low to moderate heterogeneity, and a fixed-effect model was applied; *I*^2^ > 50% indicated substantial heterogeneity, and a random-effects model was applied, with subgroup analyses or meta-regression conducted to explore potential sources of heterogeneity. Publication bias was assessed qualitatively using funnel plots, and if ≥10 studies were included, Egger’s test was performed for quantitative assessment. Sensitivity analyses were conducted by sequentially omitting individual studies to evaluate the robustness of pooled effect estimates. All tests were two-sided, and *p* < 0.05 was considered statistically significant.

## Results

### Study selection

**Figure 1:**
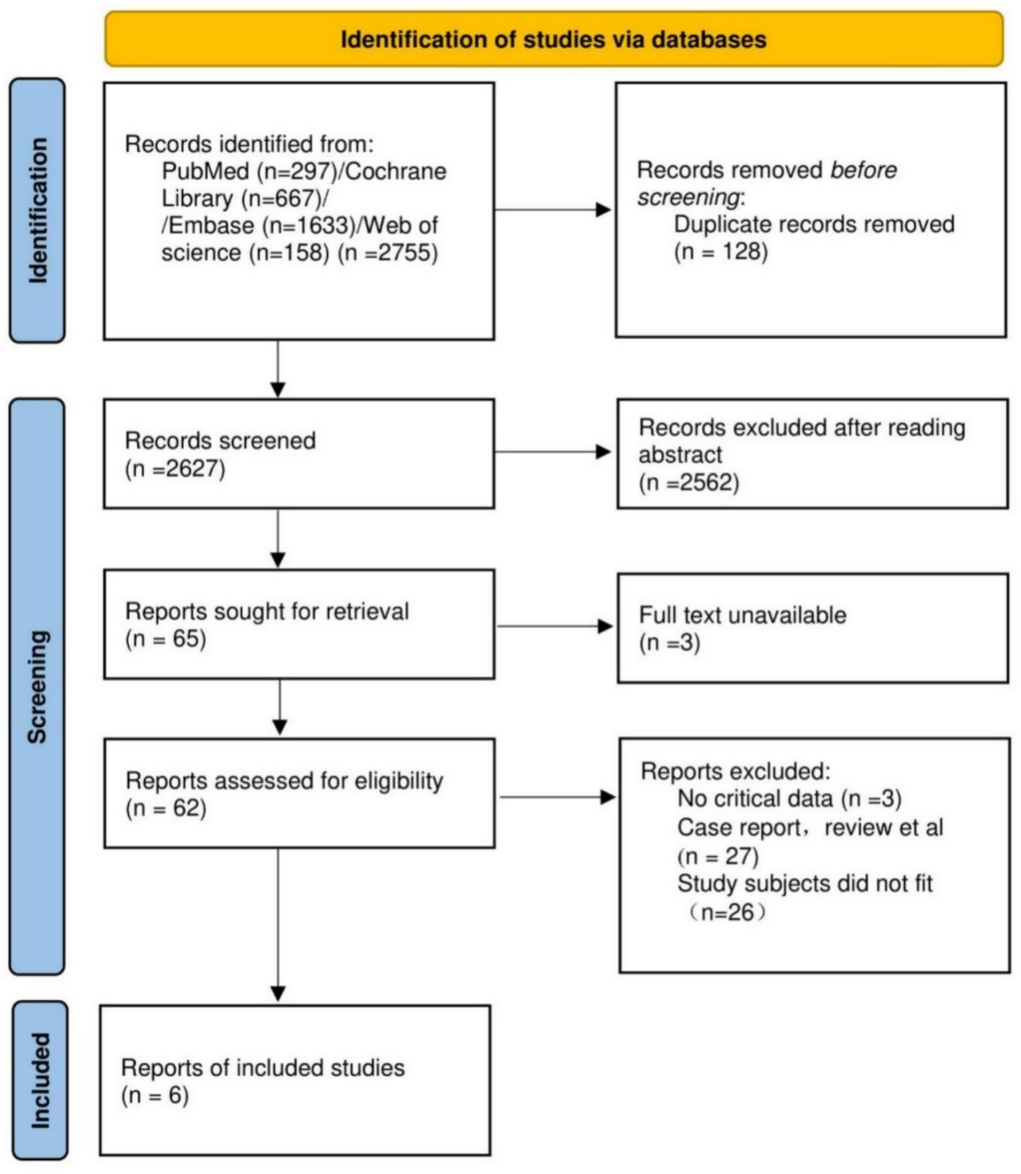
PRISMA flow diagram of study selection process.

A total of 2,755 records were identified through database searching, including 297 from PubMed, 667 from Cochrane Library, 1,633 from Embase, and 158 from Web of Science. After removing duplicates, 2,627 records remained. Following title and abstract screening, 2,562 irrelevant records were excluded, and 65 full-text articles were assessed for eligibility. During full-text assessment, 59 articles were excluded. Ultimately, 6 studies were included in the meta-analysis. Additionally, manual screening of reference lists of included studies yielded no additional eligible studies. The study selection process is presented in Figure 1.

### Characteristics of included studies

A total of six randomized controlled trials were included in this meta-analysis (13–17, 19), published between 2004 and 2023. Sample sizes ranged from 63 to 5,882 participants. The basic characteristics of each included study are presented in Table 1.

**Table 1:** Characteristics of included studies.

Study	Country	Sample size (I/C)	Patient population	Intervention	Comparator	Intervention duration	Follow-up time points	Main outcome measures
Markle-Reid et al. (2023) (13)	Canada	44/46	Stroke patients aged ≥55 years with multimorbidity, discharged home	TCSI: telephone follow-up within 2 days + virtual visits over 6 months + interdisciplinary team coordination	Usual outpatient stroke rehabilitation	6 months	6 months	SF-12 PCS (physical function) SSSMQ (self-management) P3CEQ (patient experience)
van den Berg et al. (2016) (14)	Australia	31/32	Hospitalized stroke patients planned for early supported discharge	Caregiver-mediated exercises + e-Health support (rehabilitation app + video conferencing)	Usual rehabilitation care	8 weeks	8 weeks, 12 weeks	SIS mobility NEADL (ADL) Readmission rate
Boter and HESTIA Study Group (2004) (15)	Netherlands	263/273	First-ever stroke or TIA, discharged home	Nurse-led outreach program: 3 telephone calls + 1 home visit after discharge	Usual care	5 months	6 months	SF-36 (QoL) SASC-19 (dissatisfaction with care)
Duncan et al. (2020) (16)	USA	2689/3193	Acute stroke or TIA patients, discharged home	COMPASS model: telephone follow-up within 2 days + clinic visit at 7–14 days post-discharge + individualized care plan	Usual care	90 days	90 days	SIS-16 (physical function) Blood pressure monitoring Readmission rate
Allen et al. (2009) (17)	USA	190/190	Ischemic stroke patients with NIHSS ≥1, discharged home	Comprehensive post-discharge care management: home visit + telephone follow-up + interdisciplinary team care coordination	Usual care (enhanced discharge planning)	6 months	6 months	SS-QOL (QoL) CES-D (depression) Blood pressure control
Linder et al. (2015) (19)	USA	51/48	Stroke patients <6 months post-stroke with limited access to conventional rehabilitation	Robot-assisted telerehabilitation + home exercise program vs. home exercise program alone (Note: both groups received remote interventions)	Home exercise program	8 weeks	8 weeks	SIS (QoL) CES-D (depression)

I/C, intervention/control; QoL, quality of life; ADL, activities of daily living; TIA, transient ischemic attack; NIHSS, National Institutes of Health Stroke Scale; SIS, Stroke Impact Scale; NEADL, Nottingham Extended Activities of Daily Living; CES-D, Center for Epidemiologic Studies Depression Scale; SS-QOL, Stroke-Specific Quality of Life Scale; SF-36, Short Form-36; SF-12, Short Form-12; SSSMQ, Southampton Stroke Self-Management Questionnaire; P3CEQ, Person-Centered Coordinated Care Experience Questionnaire.

### Risk of bias assessment

**Figure 2:**
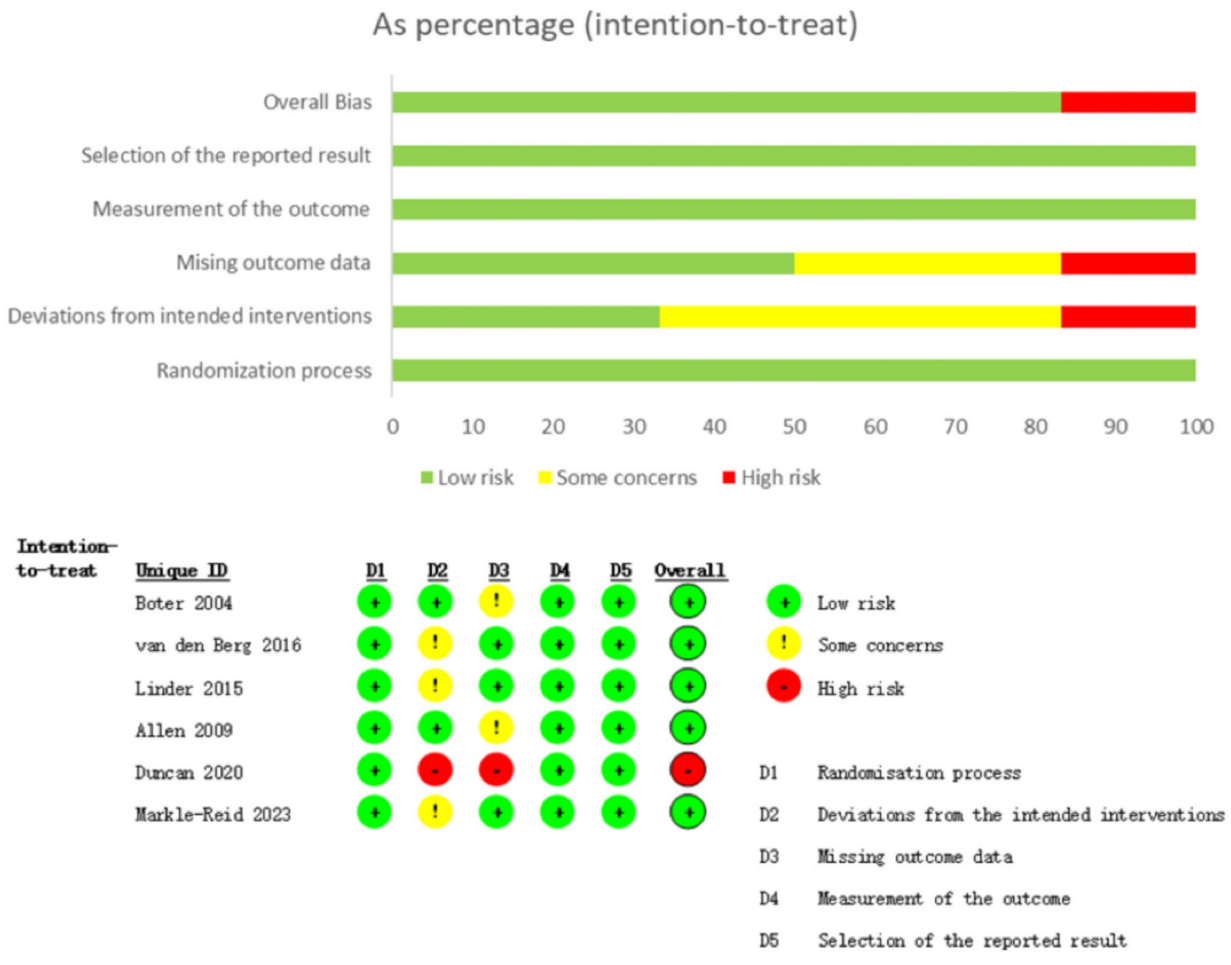
Risk of bias graph.

The methodological quality of the six included randomized controlled trials was assessed using the Cochrane ROB 2.0 tool, with results presented in Figure 2. The risk of bias assessments across the five domains for each study are detailed below.

### Meta-analysis results

#### Quality of life

**Figure 3:**
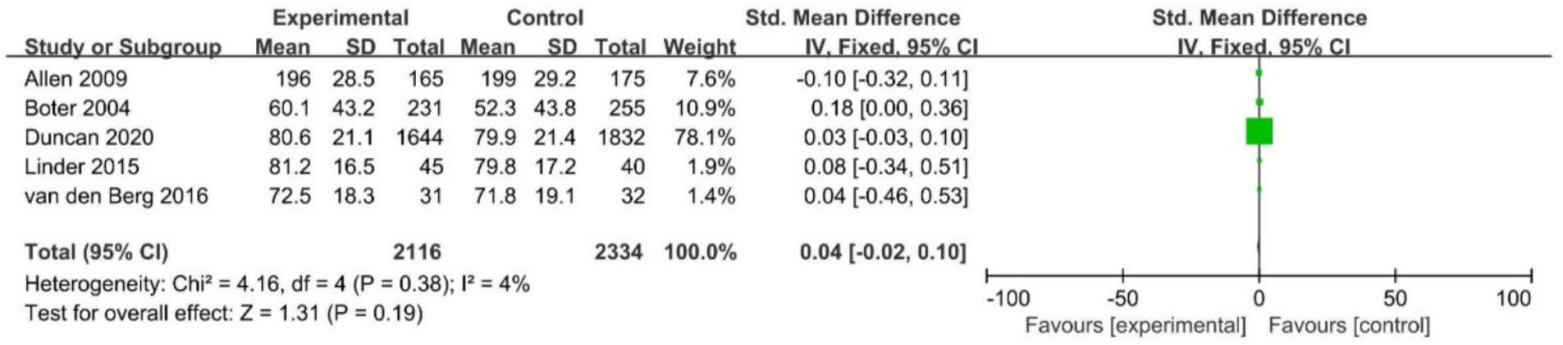
Forest plot of quality of life.

A total of five studies (14–17, 19) assessed the effect of remote transitional care on quality of life in stroke patients. Due to the use of different measurement instruments across studies, the standardized mean difference was used as the effect measure for meta-analysis. Heterogeneity testing showed low heterogeneity among studies (*I*^2^ = 4%, *p* = 0.38), and a fixed-effect model was applied. The pooled results showed no significant difference in quality of life between the remote transitional care group and the usual care group (SMD = 0.04, 95% CI: −0.02 to 0.10, *p* = 0.19). The forest plot (Figure 3) visually presents the effect sizes of individual studies and the pooled estimate.

#### Depressive symptoms

**Figure 4:**
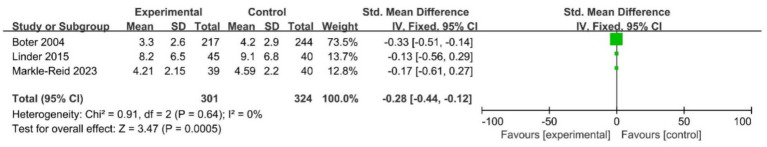
Forest plot of depressive symptoms.

Three studies (13, 15, 19) reported the effect of remote transitional care on depressive symptoms in stroke patients. The measurement instruments used across studies were different; therefore, the standardized mean difference was used for meta-analysis. Heterogeneity testing showed low heterogeneity among studies (*I*^2^ = 0%, *p* = 0.64), and a fixed-effect model was applied. The meta-analysis results demonstrated that patients in the remote transitional care group had significantly improved depressive symptoms compared with the usual care group (SMD = −0.28, 95% CI: −0.44 to −0.12, *p* = 0.0005). The forest plot (Figure 4) visually presents the effect sizes of individual studies and the pooled estimate.

#### Readmission rate

**Figure 5:**
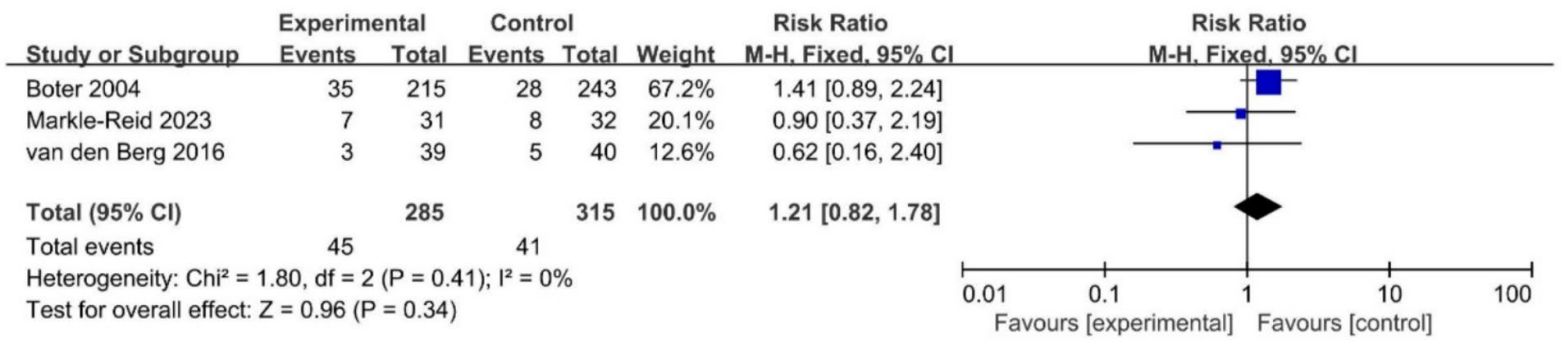
Forest plot of readmission rate.

Three studies (13–15) reported the effect of remote transitional care on readmission rates in stroke patients. Readmission rate was analyzed as a dichotomous variable, and the risk ratio was used as the effect measure for meta-analysis. Heterogeneity testing showed no heterogeneity among studies (*I*^2^ = 0%, *p* = 0.41), and a fixed-effect model was applied. The meta-analysis results showed no statistically significant difference in readmission rates between the remote transitional care group and the usual care group (RR = 1.21, 95% CI: 0.82 to 1.78, *p* = 0.34). The forest plot (Figure 5) presents the effect sizes of individual studies and the pooled estimate.

Among the three studies reporting readmission rates, only two provided information on the causes of readmission. Boter and HESTIA Study Group (15) reported that the most common reasons for hospital readmission were recurrent stroke or transient ischemic attack (29% of readmissions), cardiovascular events (21%), and infections (16%). Markle-Reid et al. (13) noted that readmissions were primarily due to stroke-related complications, falls, and exacerbations of comorbid conditions (e.g., heart failure and pneumonia); however, specific proportions were not reported. Van den Berg et al. reported readmission as a secondary outcome but did not specify reasons. The heterogeneity in reporting of readmission causes further limits the interpretability of the pooled readmission rate effect.

### Sensitivity analysis

To evaluate the robustness of the meta-analysis findings, we conducted two sensitivity analyses. First, we performed a leave-one-out analysis by sequentially omitting each individual study to assess its impact on the pooled effect size. Second, given that the study by Duncan et al. (16) was rated as high risk of bias in the quality assessment, we repeated the meta-analysis after excluding this study to evaluate its influence on the overall results.

The sensitivity analyses revealed that for all three primary outcomes (quality of life, depressive symptoms, and readmission rate), the direction, statistical significance, and heterogeneity of the pooled effect sizes did not change materially after sequentially omitting any single study. After excluding the high-risk study by Duncan et al. (16), the meta-analysis results for each outcome remained consistent with the primary analyses. These findings suggest that the meta-analysis results were robust and not excessively influenced by any individual study, supporting the credibility of the main conclusions.

## Discussion

This meta-analysis systematically evaluated the effects of remote transitional care on stroke patients, including six randomized controlled trials with over 4,000 participants. The results demonstrated that remote transitional care significantly improved depressive symptoms in stroke patients (SMD = −0.28, 95% CI: −0.44 to −0.12, *p* = 0.0005). However, no significant benefits were observed for quality of life (SMD = 0.04, 95% CI: −0.02 to 0.10, *p* = 0.19) or readmission rates (RR = 1.21, 95% CI: 0.82 to 1.78, *p* = 0.34). Sensitivity analyses confirmed the robustness of these findings, indicating that they were not excessively influenced by any individual study.

This meta-analysis did not find a significant improvement in quality of life with remote transitional care, a finding consistent with previous study (20) but divergent from others (21). Several factors may explain this null finding. First, the included studies used different instruments to measure quality of life; although we used the standardized mean difference to pool results, differences in measurement dimensions and sensitivity across scales may have affected the accuracy of the pooled estimate. Second, follow-up time points for quality of life varied across studies (ranging from 8 weeks to 12 months), and improvements in quality of life may require longer follow-up to become apparent. Third, in the study by Boter et al. (15), control group patients received relatively intensive post-discharge usual care, which may have diluted the relative benefit of the intervention. Notably, although the pooled effect was not statistically significant, individual studies by Markle-Reid et al. (13) and van den Berg et al. (14) demonstrated positive effects on quality of life, suggesting that specific forms of remote intervention or particular patient subgroups may derive greater benefit. Future research should explore more sensitive stroke-specific quality of life measures and extend follow-up duration to comprehensively assess intervention effects.

The finding that remote transitional care significantly improved depressive symptoms in stroke patients is consistent with previous research. Markle-Reid et al. (13) reported that a 6-month transitional care intervention reduced depression scores. Although the difference did not reach statistical significance, this trend was consistent with our findings. Van den Berg et al. (14) also found that caregiver-mediated telerehabilitation improved caregiver self-efficacy and fatigue, indirectly supporting the positive psychological impact of remote interventions. The improvement in depressive symptoms may be attributed to several mechanisms. First, regular telephone follow-up and remote support provide patients with continuous emotional connection, reducing feelings of isolation and abandonment after discharge (22). Second, health education components within remote interventions help patients better understand their condition and self-management, enhancing confidence in coping with the disease (13). Additionally, early identification and intervention for depressive symptoms may prevent further deterioration of depression (16, 22). These findings suggest that remote transitional care can serve as an effective adjunctive approach for managing post-stroke depression.

Regarding readmission rates, this meta-analysis did not find that remote transitional care significantly reduced the risk of hospital readmission in stroke patients. This finding is consistent with the individual study results of Boter et al. (15) and Duncan et al. (16), both of which reported no significant reduction in readmission rates with remote transitional care. However, the per-protocol analysis by van den Berg et al. (14) demonstrated that patients who received the full telerehabilitation intervention had significantly fewer readmissions over 12 months, suggesting that intervention adherence may be a critical factor influencing readmission outcomes. In the COMPASS trial by Duncan et al. (16), although the ITT analysis showed no improvement in readmission rates, post-hoc analysis revealed better functional outcomes among patients who actually received the intervention. Several explanations may account for the null finding for readmission rates. First, readmission as a clinical outcome is influenced by multiple factors, including patients’ underlying comorbidities, social support systems, and healthcare accessibility; a single intervention may be insufficient to comprehensively modify these complex factors (23). Second, definitions of readmission and follow-up periods varied across included studies, potentially increasing heterogeneity in the pooled analysis. Third, with improvements in acute stroke care quality, short-term readmission rates after stroke are already relatively low, leaving limited room for further reduction (24). Future research should focus on high-risk subgroups (e.g., older adults, patients with multimorbidity) and adopt standardized definitions of readmission and follow-up periods.

Heterogeneity testing revealed varying results across outcomes in this meta-analysis. The pooled analyses for depressive symptoms and readmission rates showed low heterogeneity (*I*^2^ = 0%), indicating consistent findings across studies for these outcomes. The pooled analysis for quality of life also demonstrated low heterogeneity (*I*^2^ = 4%), suggesting homogeneity among study results. Regarding methodological quality, five studies were rated as having “some concerns” overall, while one study was rated as “high risk.” Although the study by Duncan et al. (16) had the largest sample size, its evidence strength was compromised by low intervention fidelity (only 35% of patients received the full intervention) and high rates of missing outcome data. Sensitivity analyses showed that excluding this high-risk study did not materially change the pooled effect estimates for any outcome, suggesting that our findings are relatively robust.

This study has several limitations. First, the number of included studies was relatively small (only six), and there was considerable variation across studies in intervention content, intensity, and duration, which may affect the interpretation of pooled results. Second, incomplete data reporting in some studies—such as Boter et al. (15) reporting medians rather than means, and Markle-Reid et al. (13) reporting mean differences rather than group-specific values—required estimation methods that may have introduced bias. Third, the study by Duncan et al. (16) had a high risk of bias, and although sensitivity analyses suggested limited impact on overall results, findings should be interpreted with caution. Fourth, due to the limited number of included studies, we were unable to conduct comprehensive subgroup analyses (e.g., by intervention type, patient age, stroke severity) or publication bias assessment (funnel plot symmetry testing requires ≥10 studies). Fifth, all included studies were conducted in high-income countries (Netherlands, United States, Australia, Canada), limiting the generalizability of findings to low- and middle-income countries.

This study has important implications for clinical practice and future research. First, remote transitional care significantly improves depressive symptoms in stroke patients, suggesting that routine post-discharge care should incorporate remote psychological support components, such as regular telephone follow-up and emotional assessment. Second, the number of studies contributing to each outcome was imbalanced: five studies reported quality of life, whereas only three studies each reported depressive symptoms and readmission rates. This imbalance, together with the reliance on published aggregated data rather than individual participant-level data, introduces potential selection bias and limits the precision of our effect estimates. The small number of studies for depressive symptoms and readmission rates precluded meaningful subgroup analyses or formal publication bias assessment (e.g., funnel plot symmetry testing), and the use of summary statistics may have reduced statistical power to detect smaller but clinically important effects. Third, quality of life as a multidimensional concept may require more comprehensive interventions and longer follow-up to observe improvement; future research should focus on the application of stroke-specific quality of life measures. Fourth, reduction in readmission rates may require higher-intensity interventions and better patient adherence; the study by van den Berg et al. suggests that ensuring complete intervention receipt may be key to achieving clinical benefits. Furthermore, the assessment of depressive symptoms was not standardized across studies: different cutoff scores on the CES-D were used, and none of the three studies included in the depressive symptoms meta-analysis reported severity grading (mild/moderate/severe). Moreover, none of the included studies applied formal diagnostic criteria for post-stroke depression (e.g., DSM-5), which limits the clinical interpretability of the meta-analysis findings on depressive symptoms. Similarly, reasons for hospital readmission were inconsistently reported, with only two of three studies providing partial data on readmission causes. These methodological inconsistencies across primary studies constrain our ability to draw precise conclusions regarding depression severity and specific drivers of readmission. Future research directions include: conducting larger multicenter pragmatic randomized controlled trials to explore the comparative effectiveness of different remote intervention modalities (e.g., telephone follow-up, video rehabilitation, mobile health applications); in particular, head-to-head trials directly comparing telephone-only versus video-based versus hybrid approaches are needed to determine whether specific modalities confer incremental benefits for depressive symptoms, quality of life, or readmission outcomes; tailoring interventions for specific subgroups (e.g., older adults, patients with multimorbidity, rural residents); adopting standardized outcome measures and uniform follow-up periods to facilitate cross-study comparisons and pooling; and conducting health economic evaluations to assess the cost-effectiveness of remote transitional care.

Although all included studies were conducted in high-income Western nations, the findings of this meta-analysis may offer valuable lessons for low- and middle-income countries (LMICs), where stroke burden is disproportionately high and healthcare resources are often constrained. Several considerations are relevant when translating remote transitional care to LMIC settings. First, the significant improvement in depressive symptoms observed in our meta-analysis suggests that even low-intensity remote interventions (e.g., regular telephone follow-ups, brief psychosocial support) could be implemented at relatively low cost in LMICs, where access to mental health services is particularly limited. Second, the null findings for quality of life and readmission rates indicate that simple remote follow-up may be insufficient in LMICs; context-specific adaptations—such as integrating community health workers, using text messages or voice calls rather than video-based platforms, and addressing social determinants of health—are likely necessary to achieve meaningful improvements. Third, infrastructure challenges (e.g., inconsistent internet connectivity, lack of reliable electricity in rural areas) and the digital divide (lower smartphone ownership and health literacy among older stroke patients) must be addressed through hybrid models that combine remote and in-person care, as well as investments in basic telecommunication infrastructure. Fourth, task-shifting to nurses or community health workers, coupled with mHealth applications optimized for low-bandwidth environments, represents a promising and affordable strategy for scaling up remote transitional care in LMICs. Future research in these settings should prioritize pragmatic implementation trials that evaluate not only clinical effectiveness but also cost-effectiveness, feasibility, acceptability, and equity impact, with the goal of developing locally adaptable, evidence-based remote transitional care models for stroke patients.

## Conclusion

In conclusion, this meta-analysis suggests that remote transitional care significantly improves depressive symptoms in stroke patients, although no significant benefits were observed for quality of life or readmission rates. However, these findings are subject to important limitations, including the small number of included studies for depressive symptoms and readmission outcomes (only three each), the imbalance in study contributions across outcomes, and the reliance on aggregated published data rather than individual participant data. The findings are relatively robust but are limited by the small number of included studies and variations in methodological quality. Future high-quality, large-sample randomized controlled trials with standardized outcome reporting and individual participant data access are needed to further validate the effectiveness of remote transitional care and to explore optimal intervention modalities, intensity, and implementation strategies to maximize clinical benefits for stroke patients.

## Data Availability

The original contributions presented in the study are included in the article/supplementary material, further inquiries can be directed to the corresponding author.
